# Self-expandable metallic stents as a bridge to surgery in obstructive right- and left-sided colorectal cancer: a multicenter cohort study

**DOI:** 10.1038/s41598-023-27767-1

**Published:** 2023-01-09

**Authors:** Eui Myung Kim, Jun Ho Park, Byung Chun Kim, Il Tae Son, Jeong Yeon Kim, Jong Wan Kim

**Affiliations:** 1 Department of Surgery, Dongtan Sacred Heart Hospital, Hallym University College of Medicine, 40, Sukwoo-Dong, Hwaseong-Si, Gyeonggi-Do 445-170 Republic of Korea; 2 Department of Surgery, Kangdong Sacred Heart Hospital, Hallym University College of Medicine, 445 Gil-1-dong, Gangdong-gu, Seoul, 134-701 Republic of Korea; 3 Department of Surgery, Kangnam Sacred Heart Hospital, Hallym University College of Medicine, 948-1, 1, Shingil-ro, Yeongdeungpo-gu, Seoul, 150-950 Republic of Korea; 4 Department of Surgery, Hallym Sacred Heart Hospital, Hallym University College of Medicine, Anyang Si, 445-907 Republic of Korea

**Keywords:** Gastroenterology, Medical research, Oncology

## Abstract

The insertion of a self-expandable metal stent (SEMS) has been proposed as an alternative to emergent surgery (ES) for obstructive colorectal cancer (CRC). We aimed to evaluate the perioperative and oncologic outcomes of SEMS as a bridge to surgery in obstructive CRC, as compared with ES. We retrospectively reviewed the medical records of patients who underwent curative resection of obstructive CRC at four Hallym University-affiliated hospitals between January 2010 and December 2019. All patients were analyzed overall colon, then according to the side of obstruction (overall, right or left). Of 167 patients, 52 patients underwent ES and 115 underwent SEMS insertion and surgery (SEMS group). The postoperative hospital stay and time to soft diet were shorter in the SEMS group than in the ES group for overall and both sided cancer. The SEMS group had lower rates of stoma formation and severe complications for overall and for left-sided cancer. The 5-year overall survival (*P* = 0.682) and disease-free survival (*P* = 0.233) rates were similar in both groups. SEMS insertion as a bridge to surgery was associated with faster recovery, a lower rate of stoma formation with similar oncologic outcomes to those of ES.

## Introduction

Colorectal cancer (CRC) is one of the most common cancers worldwide, and its incidence has increased in Korea in recent decades^1,2^. Between 8.3 and 16.3% of patients with CRC present with obstructive symptoms at the time of diagnosis^3–6^. Although patients with (malignant) colonic obstruction usually undergo emergency surgery (ES), this procedure is associated with high morbidity (38–41.2%) and mortality (8.2–14.8%)^6–11^.

In recent years, the insertion of a self-expandable metal stent (SEMS) has been proposed as an alternative to ES for obstructive CRC. Since Dohomoto et al. first reported the placement of a SEMS in 1991^12^, stents have been used for curative decompression as a bridge to elective surgery and for palliative treatment of unresectable CRC obstruction. Prior studies have reported better clinical outcomes of SEMS placement compared with ES, including lower postoperative morbidity, lower rate of stoma formation, and shorter postoperative hospital stay^13–17^. Moreover, stent insertion may allow surgeons to perform minimally invasive surgery (MIS) in patients with CRC obstruction^18^.

However, there is still some concern about stent-related complications, including perforation, migration, and obstruction^8^. SEMS insertion could result in bowel perforation, leading to the dissemination of tumor cells^18–20^. The 2020 European Society of Gastrointestinal Endoscopy guidelines recommend SEMS insertion as a bridge to elective surgery as an alternative to ES in patients with potentially curable right-sided and left-sided obstructive colon cancer and as the preferred palliative treatment for obstructing colon cancer^21^. According to the recent American Society of Colon and Rectum Surgeons guidelines, the initial treatment choice for patients with curable and obstructing left-side cancer, including stent insertion, diverting colostomy with interval colectomy, or initial colectomy with consideration of the oncologic safety, should be based on patient factors and the institution’s expertise. For patients with curable and obstructing right or transverse colon cancer, initial colectomy or tent insertion with subsequent interval colectomy may be performed^22^.

The purpose of this study was to evaluate the perioperative and oncologic outcomes of SEMS as a bridge to surgery in patients with obstructive CRC, as compared with ES.

## Methods

We reviewed the medical records of patients who underwent curative resection of obstructive CRC cancer at four Hallym University-affiliated hospitals between January 2010 and December 2019. The Institutional Review Board of Hallym approved the study (approval number 2020-11-001) and complies with the Helsinki Declaration. Due to the retrospective nature of the study, the Institutional Review Board waived the need to obtain informed consent.

We excluded patients with incomplete medical records, a history of familial adenomatous polyposis syndrome or Lynch syndrome, colon perforation, or hemodynamic instability at the time of diagnosis, as well as those with mid to lower rectal cancer because the level of stenting was too low. We also excluded patients who underwent palliative surgery due to unresectable metastasis. The eligible patients were divided into two groups depending on whether they underwent SEMS or ES. There were no definite guidelines for the treatment of colonic obstruction. The initial treatment was selected during multidisciplinary meetings or consultations among surgeons, gastroenterologists, and radiologists. Stent insertion was attempted in patients without signs of peritoneal irritation indicative of perforation or peritonitis. The SEMS group comprised patients who underwent colonic stenting followed by elective surgery (within about 2 weeks). ES was performed in the event of technical or clinical failure of colonoscopy or stenting. These patients were allocated to SEMS group. After confirming the SEMS was correctly positioned on a plain X-ray, the patients were carefully monitored to detect potential symptoms such as abdominal distension or pain. ES was initially performed if the tumor was too large to be covered with a stent, if there was a high risk of perforating the tumor during the stenting procedure (e.g., due to acute angulation), or if the patient’s vital signs were unstable. The ES group comprised patients who underwent emergency surgery within 24 h of admission to hospital. The type and extent of surgery was determined at the surgeon’s discretion, based on the patient’s clinical condition, intraoperative findings, and their experience.

Colonic obstruction was defined based on clinical symptoms, including abdominal distension, constipation, and vomiting, together with radiologic evidence, such as severe dilation of the proximal colon from a suspicious obstructive lesion on plain abdominal X-ray and abdominal computed tomography (CT), or an inability to pass an endoscope through the obstructive lesion. Right-sided cancers were defined as those located between the cecum to the transverse colon, and left-sided cancers were defined as cancers located between the splenic flexure and upper rectum (up to 15 cm from the anal verge).

Clinical success was defined as the relief of obstructive symptoms by gas and stool passage within 48 h after stent placement. Technical success was defined as the correct placement and deployment of the stent at the stenotic site. The severity of postoperative complications was classified using the Clavien–Dindo classification^23^.

Overall survival (OS) was defined as the time from surgery to death from any cause or the time to the last follow-up visit. Disease-free survival (DFS) was defined as the time from surgery to recurrence or death from any cause. Patients without recurrence were censored at the date of their last follow-up visit.

### Statistical analysis

All statistical analyses were performed with SPSS version 24.0 (IBM, Armonk, NY, USA). Continuous variables are presented as the mean and standard deviation and were compared using the Mann–Whitney U test. Categorical variables were analyzed using Fisher’s exact test and are presented as the number and percent of patients. The OS and DFS rates were calculated using the Kaplan–Meier method and differences were evaluated using log-rank tests. P values of < 0.05 were considered statistically significant.

## Results

The present study included 167 patients with obstructive CRC treated over a 10-year period (between January 2010 and December 2019). Of these, 115 (68.9%) underwent SEMS insertion and 52 (31.1%) underwent ES. Stent insertion was technically successful in 107 of 115 patients (92.6%). The technical failures in eight patients were due to the impossibility of introducing the guidewire through the malignant lesion in six patients and stent migration in two patients. The clinical success rate was 90.6% (97/107). Of 10 patients with clinical failure, five required ES due to colon perforation, and one of these patients died because of sepsis. Elective radical surgery was performed 15.5 days after stent insertion. Of 115 patients in SEMS group, 54 patients (46.9%) discharged after stent insertion and re-admission for surgery.

Table 1 presents the patients’ characteristics. The median age of the SEMS group was lower than that of the ES group (65.2 years vs. 69.1 years), although this was not statistically significant (*P* = 0.078). Gender, body mass index, and American Society of Anesthesiologists score were similar in both groups. The tumors in the SEMS group were more frequently located in the left side than in the right side of the colon (85.2% vs. 14.8%), whereas tumors in the ES group were more frequently located in the right side than in the left side (53.4% vs. 46.2%). Owing to the difference in CRC location between the two groups, the perioperative outcomes and complications were analyzed for the left- and right-sided CRCs separately. There were no differences between the SEMS and ES groups in terms of pT, pN, stage, and histologic type. However, the rate of perineural invasion was greater in the SEMS group (45.2% vs. 21.6%, *P* = 0.005). The mean number of harvested lymph nodes was 29.1 and 31.0 in the SEMS and ES groups, respectively (*P* = 0.492).

**Table 1:** Patient characteristics in obstructive colorectal cancer.

	SEMS (n = 115)	ES (n = 52)	*P*
Age (years)	65.2 (12.8)	69.1 (13.5)	0.078
Gender, n (%)			0.507
Female	69 (60.0)	34 (65.4)	
Male	46 (40.0)	18 (34.6)	
BMI (kg/m^2^)	22.7 (3.4)	22.1 (3.6)	0.316
ASA, n (%)			0.378
I	13 (11.3)	5 (9.6)	
II	67 (58.3)	26 (50.0)	
III	31 (27.0)	20 (38.5)	
IV	4 (3.5)	1 (1.9)	
Location			< 0.001
Right colon	17 (14.8)	28 (53.8)	
Left colon	98 (85.2)	24 (46.2)	
Comorbidities	67 (58.3)	27 (51.9)	0.445
Comorbidities ≥ 2	28 (24.3)	11 (21.2)	0.651
pT			0.847
T3	90 (78.3)	40 (76.9)	
T4	25 (21.7)	12 (23.1)	
pN			
N0	44 (38.3)	27 (51.9)	0.235
N1	42 (36.5)	16 (30.8)	
N2	29 (25.2)	9 (17.3)	
TNM stage, *n* (%)			0.152
II	44 (38.3)	27 (51.9)	
III	63 (54.8)	24 (46.2)	
IV	8 (7.0)	1 (1.9)	
Histologic type			0.466
Well	14 (12.2)	17 (32.7)	
Moderate	98 (85.2)	29 (55.8)	
Poorly	2 (1.7)	3 (5.8)	
Mucinous	1 (0.9)	3 (5.8)	
Lymphovascular invasion	70 (60.9)	30 (57.7)	0.698
Perineural invasion	52 (45.2)	11 (21.6)	0.005
Number of harvested LN	29.1 (15.1)	31.0 (19.1)	0.492

Data are presented as the number of patients (%) or mean (standard deviation) unless otherwise stated.

*SEMS* self-expandable metallic stent, *ES* emergent surgery, *n* number, *BMI* body mass index, *ASA* American Society of Anesthesiologists, *LN* lymph nodes.

Table 2 shows the perioperative outcomes. The diversion rate was greater in the ES group (38.4% vs 20.9%, *P* = 0.017), and MIS was more frequently performed in the SEMS group (73.9% vs. 30.8%, *P* < 0.001). The open conversion rate in the SEMS and ES groups was 10.6% and 31.3%, respectively (*P* = 0.028). The diversion rate in the SEMS and ES group was 20.9% and 40.4% (*P* = 0.008) and the reversal rate was 54.2% and 42.9%, respectively (*P* = 0.449). The times to first sips of water (3.7 vs. 4.7 days, *P* = 0.018) and soft diet (5.4 vs. 7.2 days, *P* = 0.002) intake, and the postoperative hospital stay (12.3 vs. 16.7 days, *P* = 0.017) were shorter in the SEMS group than in the ES group. Although the 30-day mortality rate was similar, the reoperation rate was greater in the ES group than in the SEMS group (7.7% vs. 0.9%, *P* = 0.033).

**Table 2:** Perioperative outcomes in obstructive colorectal cancer.

	SEMS (n = 115)	ES (n = 52)	*P*
Operation time (min)	230.24 (80.3)	207.4 (90.6)	0.104
Diversion	24 (20.9)	20 (38.4)	0.017
Ileostomy	15 (13.0)	11 (21.1)	
Colostomy	9 (7.8)	9 (17.3)	
Diversion reversal	13 (54.2)	9 (42.9)	0.449
MIS	85 (73.9)	16 (30.8)	< 0.001
Open conversion	9 (10.6)	5 (31.3)	0.028
Time to sips of water (days)	3.7 (1.6)	4.7 (2.6)	0.018
Time to soft food intake (days)	5.4 (2.1)	7.2 (3.5)	0.002
Duration of POD (days)	12.3 (7.8)	16.7 (14.7)	0.017
Reoperation	1 (0.9)	4 (7.7)	0.033
Mortality within 30 days	2 (1.7)	3 (5.8)	0.175
Adjuvant chemotherapy	77 (67.0)	29 (55.8)	0.164
5FU + LV	15 (13.0)	3 (5.8)	
FOLFOX/FOLFIRI	55 (44.8)	22 (42.3)	
XELODA/XELOX	7 (6.0)	4 (7.7)	

Data are presented as the number of patients (%) or median (standard deviation) unless otherwise stated.

*SEMS* self-expandable metallic stent, *ES* emergent surgery*, n* number*, MIS* minimal invasive surgery*, POD* postoperative hospital days.

The postoperative complications in obstructive CRC are listed in Table 3. The complication rate was similar between the SEMS and ES groups (20.0% vs. 30.8%, *P* = 0.128). Pneumonia and ileus each occurred in nine patients, followed by pneumonia, wound infection, intraabdominal abscess, and anastomotic leakage. Using the Clavien–Dindo classification, the rate of Grade III–V complications in patients was greater in the ES group than in the SEMS group (17.3% vs. 5.2%, *P* = 0.011).

**Table 3:** Postoperative complications in obstructive colorectal cancer.

	SEMS (n = 115)	ES (n = 52)	*P*
Complications	23 (20.0)	16 (30.8)	0.128
Wound infection	4 (3.5)	2 (3.8)	
Ileus	4 (3.5)	5 (9.6)	
Intrabdominal abscess	1 (0.9)	0 (0.0)	
Anastomotic leakage	2 (1.7)	1 (1.9)	
Pneumonia	6 (5.2)	3 (5.8)	
Others (medical)	8 (7.0)	9 (17.3)	
Complications ≥ 2	4 (3.5)	6 (11.5)	0.072
Clavien-Dindo classification			0.011
I/II	17 (14.7)	7 (13.4)	
III/IV/V	6 (5.2)	9 (17.3)	

Data are presented as the number of patients (%) or median (standard deviation) unless otherwise stated.

*SEMS* self-expandable metallic stent, *ES* emergent surgery*, n* number.

Table 4 shows the perioperative outcomes according to the affected side. The diversion rate was greater in the ES group than in the SEMS group in patients with left-sided cancer (66.7% vs. 23.4%, P < 0.001). The rate of MIS was greater in the SEMS group than in the ES group for right-sided cancer (70.6% vs. 21.4%, *P* = 0.001) and left-sided cancer (74.5% vs. 41.7%, *P* = 0.002). For both sides, postoperative hospital stay (SEMS group vs. ES group; right-sided cancer: 10.4 days vs. 13.6 days, *P* = 0.037; left-sided cancer: 12.8 days vs. 18.3 days, *P* = 0.038) and time to resuming a soft diet (right-sided cancer: 5.5 days vs. 7.4 days, *P* = 0.019; left-sided cancer: 5.5 days vs. 7.0 days, *P* = 0.016) were shorter in the SEMS group than in the ES group. Moreover, for right-sided cancer, the time to the first sips of water was shorter in the SEMS group than in the ES group (3.5 days vs. 4.8 days, *P* = 0.038). According to the Clavien–Dindo classification, the rate of Grade III–V complications was greater in the ES group than in the SEMS group for left-sided cancer (20.8% vs. 6.1%, *P* = 0.024). The reoperation rate and 30-day mortality rate were similar in both groups.

**Table 4:** Perioperative outcome according to location of colorectal cancer.

	Right			Left		
	SEMS (n = 17)	ES (n = 28)	*P*	SEMS (n = 98)	ES (n = 24)	*P*
Diversion	1 (5.9)	4 (14.3)	0.635	23 (23.4)	16 (66.7)	< 0.001
Ileostomy	1 (5.9)	4 (14.3)		14 (14.3)	7 (29.2)	
Colostomy	0 (0.0)	0 (0.0)		9 (9.2)	9 (37.5)	
MIS	12 (70.6)	6 (21.4)	0.001	73 (74.5)	10 (41.7)	0.002
Open conversion	1 (8.3)	1 (16.7)	1.000	8 (11.0)	4 (40.0)	0.034
Time to SOW (days)	3.5 (1.3)	4.8 (2.3)	0.038	3.7 (1.6)	4.5 (2.8)	0.268
Time to SD intake (days)	5.5 (1.6)	7.4 (4.2)	0.019	5.5 (2.3)	7.0 (3.1)	0.016
Duration of POD (days)	10.4 (2.6)	13.6 (5.7)	0.037	12.8 (8.6)	18.3 (11.6)	0.038
Complications	2 (11.8)	10 (35.7)	0.096	21 (21.4)	6 (25.0)	0.706
Clavien-Dindo Classification ≥ 3	0 (0.0)	4 (14.3)	0.281	6 (6.1)	5 (20.8)	0.024
Reoperation	0 (0.0)	2 (7.1)	0.519	1 (1.0)	2 (8.3)	0.098
Mortality within 30 days	0 (0.0)	2 (7.1)	0.519	2 (2.0)	1 (4.2)	0.485

Data are presented as the number of patients (%) or median (standard deviation) unless otherwise stated.

*SEMS* self-expandable metallic stent, *ES* emergent surgery, *n number, MIS* minimal invasive surgery, *SOW* sips of water*, SD* soft diet*, POD* postoperative hospital days.

**Figure 1:**
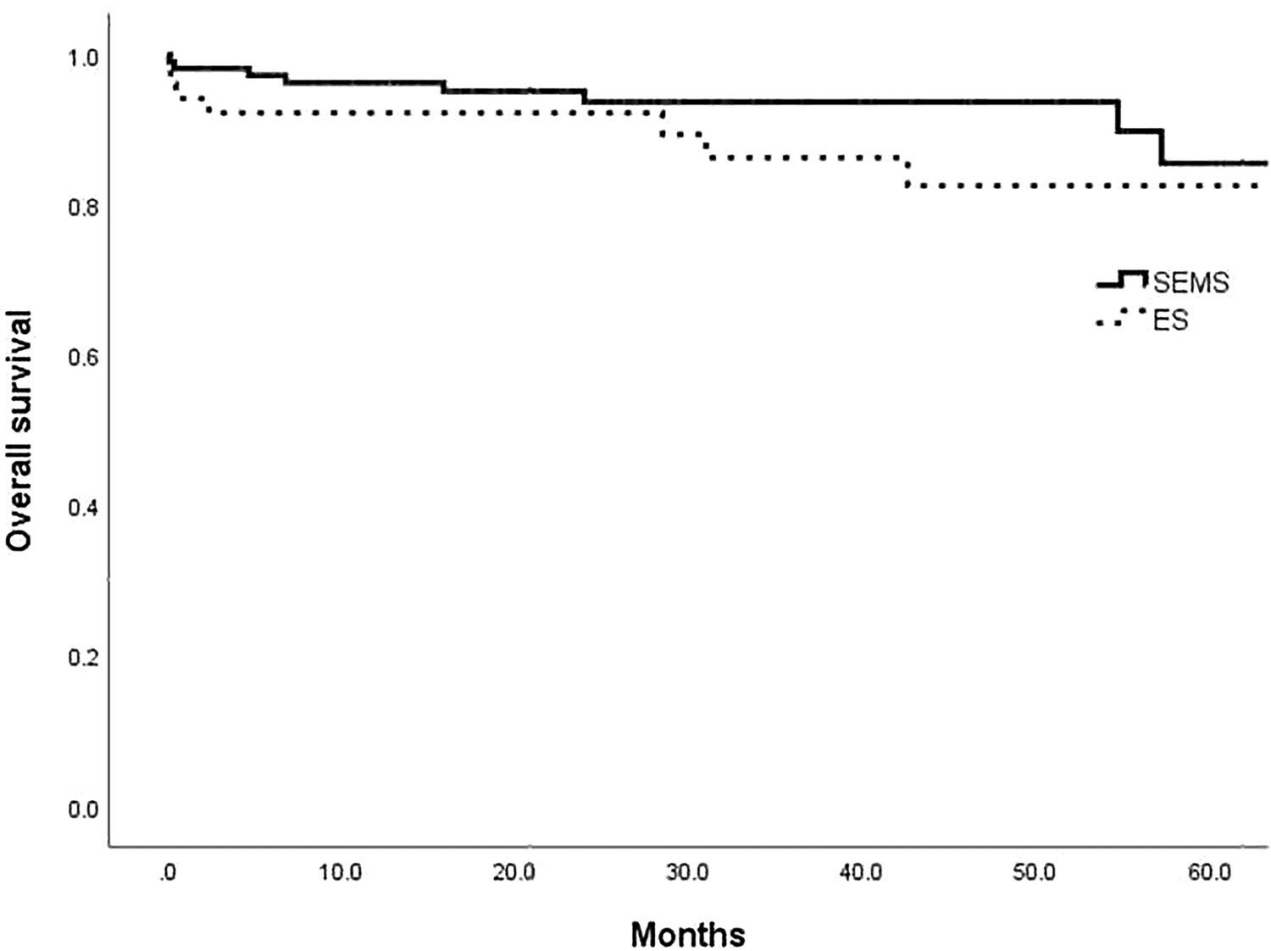
Overall survival between the SEMS and ES groups (5 years- 85.6% vs 82.6%, P = 0.682).

**Figure 2:**
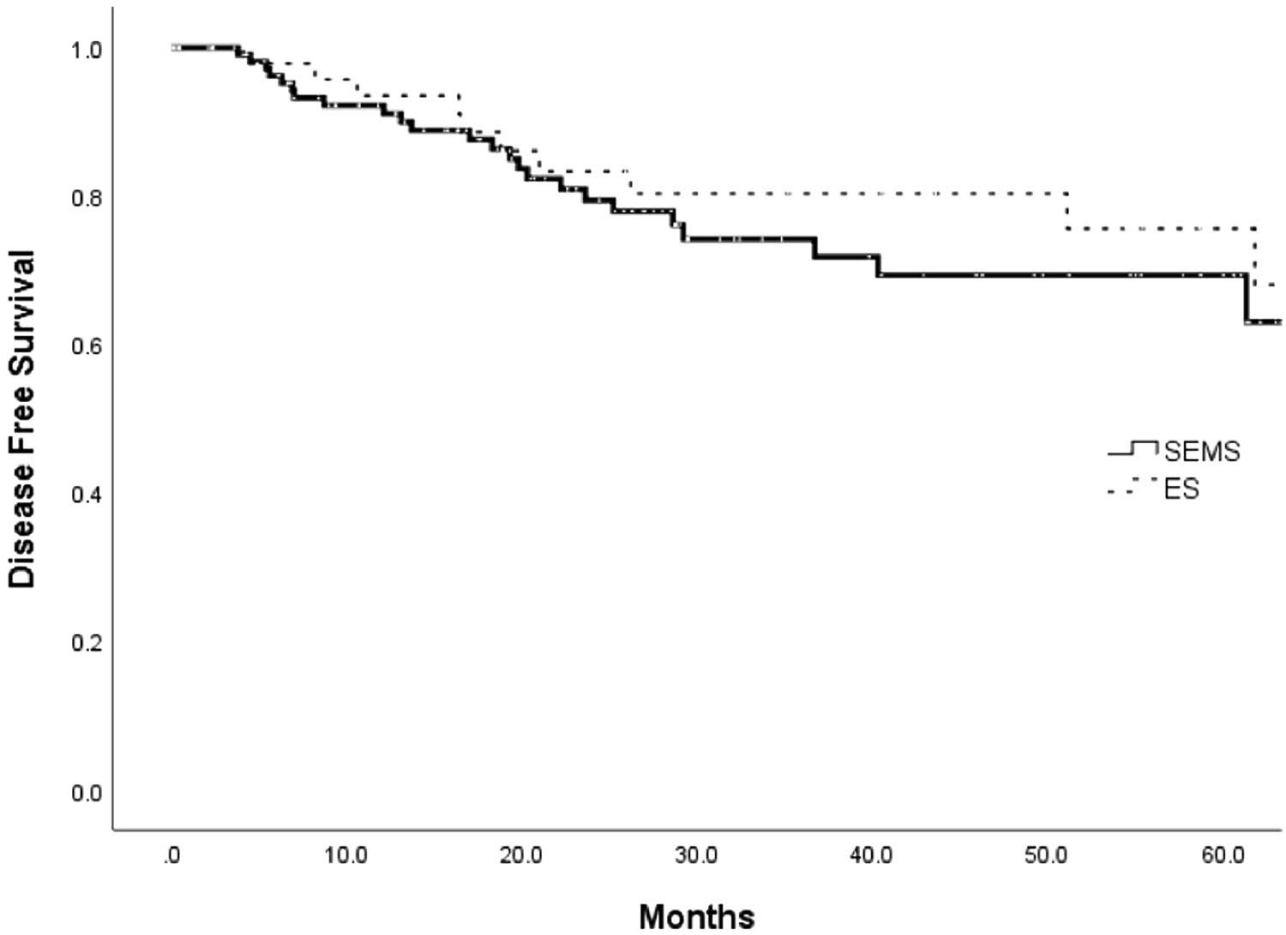
Disease-free survival between the SEMS and ES groups (5 years- 69.3% vs 75.6%, P = 0.233).

The median follow-up was 53.4 months overall (range, 3–98 months), 52.9 months in the SEMS group, and 56.3 months in the ES group. The 5-year OS rate was not significantly different between the SEMS and ES groups (85.6% vs. 82.6%, *P* = 0.682, Fig. 1). Furthermore, the 5-year DFS rate was not also significantly different between the SEMS and ES groups (69.3% vs. 75.6%, *P* = 0.328, Fig. 2). Overall, 25 patients in the SEMS group (21.7%) and 11 patients in the ES group (21.2%) experienced disease recurrence (*P* = 0.932). The most common site of recurrence was the liver, followed by the lung, peritoneum, ovary, and brain (Table 5).

**Table 5:** Distribution of tumor recurrence in obstructive colorectal cancer.

	SEMS (n = 115 )	ES (n = 52)	*P*
Recurrence, n (%)	25 (21.7)	11 (21.2)	0.932
Anastomotic site	2 (1.6)	0	
Liver	10	3	
Lung	6	4	
Peritoneum/seeding	7	1	
Ovary	3	1	
Brain	2	2	
Etc^a^	2	1	
Median follow-up (months)	52.9	56.3	

Data are presented as the number of patients (%) or median unless otherwise stated.

*SEMS* self-expandable metallic stent, *ES* emergent surgery, *n* number.

^a^Etc included Right renal vein, rectovaginal pouch, and bladder recurrence.

## Discussion

SEMS was initially proposed as palliative treatment for patients with obstructive rectal cancer in 1991. More recently, Tejero et al*.* described SEMS placement as a bridge to surgery in two cases of curable obstructive CRC^24^. Lamazza et al. reported the feasibility of using self-expandable metallic stents for treating symptomatic anastomotic leakage, anastomotic stricture, and rectovaginal fistula after colorectal resection for cancer^25–27^.

SEMS was associated with lower rates of stoma formation and postoperative complications, shorter hospital stay, and higher rates of primary anastomosis in previous studies^13–16,28,29^. A recent meta-analysis of seven randomized controlled trials showed that stent insertion could reduce stoma formation as compared with emergent resection (28.8% vs. 46.2%, P < 0.001) as well as postoperative complications (37.8% vs. 54.8%, P = 0.02) such as wound infection (8.1% vs. 15.5%, P = 0.001), with similar mortality. The rate of primary anastomosis was greater in the stent group (71.2% vs. 55.3%, P = 0.007)^11^. In the present study, patients in the SEMS group had a lower rate of stoma formation (overall: P = 0.017; left-sided cancer: P < 0.001) and faster recovery, including shorter postoperative hospital stay (overall: P = 0.017; right-sided cancer: P = 0.037; left-sided: P = 0.038) and shorter times to sips of water (overall: P = 0.018; right-sided cancer: P = 0.038) and soft diet (overall: P = 0.002; right-sided cancer: P = 0.019; left-sided cancer: P = 0.016). The rate of severe postoperative complications (Clavien–Dindo grade ≥ 3) was lower in the SEMS group than in the ES group (overall: P = 0.011; left-sided cancer: P = 0.024).

When used as a bridge to elective surgery, SEMS could also permit bowel decompression and preparation and provide sufficient time for preoperative assessment for tumor staging and stabilization of comorbidities^17,30^. These factors may contribute to the lower rates of stoma formation and complications in the SEMS groups, resulting in faster recovery.

Moreover, in patients with unresectable CRC, several studies have reported that placement of a SEMS could be considered as an initial option because it allows chemotherapy to be administered earlier than with patients undergoing surgery, and it may increase the resectability rate of metastases^28,31,32^.

Dilation of the small bowel and colon due to colonic obstruction can make it impossible to provide an adequate operation field and to manipulate laparoscopic instruments. However, MIS is possible after decompression with a metallic stent^18,33^. Kim et al. compared stenting with elective laparoscopic surgery and emergent open surgery in patients with left-sided obstructive colon cancer^18^. In the present study, MIS was more frequently performed in the SEMS group than in the ES group (overall: P < 0.001; right-sided cancer: P = 0.001; left-sided cancer: P = 0.002).

One of the major concerns of stent insertion is the risk of dissemination of tumor cells as a consequence of bowel perforation, which could convert a potentially curable cancer into an incurable cancer. The bowel could be perforated through friable tumor tissue during (potentially excessive) manipulation of the guidewire or erosion of the colon wall by the edge of the stent^34^. SEMS-related perforations occurred in 4.3% of patients in the study, similar to the rates reported in previous studies (4–12%)^29,34–37^. And one of the five patients died after perforation due to sepsis. A multivariable analysis by Kim et al.indicated that perforation was an independent risk factor for recurrence (*P* = 0.030) and peritoneal seeding (*P* = 0.016)^34^. Although it is difficult to draw a definite conclusion due to the small incidence of mortality case, care should be taken to insert the stent.

Migration and restenosis are potential complications associated with stent insertion. Park et al. reported that peritoneal carcinomatosis (*P* = 0.041) and stent type (*P* = 0.017) were significant risk factors for stent-related complications^15^. Peritoneal carcinomatosis causes multiple strictures and fixations in the intestine, increasing the possibility of incorrect stent insertion and stent-related complications such as re-obstruction and migration^38^. Moreover, migration was more frequent in patients implanted with a covered stent than in those who were implanted with an uncovered stent (18.9% vs. 1.4%)^15^.

Maruthachalam et al. reported an increase in cytokeratin-20 mRNA in peripheral blood after stent insertion and suggested the possibility that malignant tumor cells were disseminated through the stent^19^. Moreover, Yamashita et al. reported that two patients with no viable circulating tumor cells (v-CTCs) before stent insertion had an increased number of v-CTCs after stent insertion (2/4, 50%) and that two patients with v-CTCs before stent insertion also had an increased number of v-CTCs after stent insertion (2/4, 50%). These increases may be due to tumor cell dissemination into the peripheral circulation and may result in distant metastases^39^. In the present study, there were 2 recurrences at anastomotic site in the SEMS group, which might be associated with the tumor spreading through the stent.

A recent systematic review reported that stent insertion could increase the incidence of PNI, which may decrease the long-term survival of patients^40^. In the present study, although the rate of PNI was higher in the SEMS group than in the ES group (45.2% vs. 21.6%, P = 0.005), the rates were in accordance with the rates reported in previous studies that ranged from 24 to 59.1% in the stent group and from 20 to 51.4% in the ES group^40^. The authors of that review also proposed a hypothesis that stent insertion could damage the colon wall and cause tumor expansion through the damaged wall, allowing tumor cells to travel through the nerve plexus into the perineural space^40^. Meanwhile, Kim et al. reported that an interval from stent insertion to surgery of around 10–14 days is too short to influence the oncologic outcomes^18^.

Several studies and meta-analyses have compared the long-term oncologic outcomes, including OS, DFS, relapse-free survival, and disease recurrence between stent placement and emergent surgery^13,16–18,29,30,35,36^. The overall results did not indicate the superiority of either strategy. In the present study, the oncologic outcomes were similar between the SEMS and EM groups in terms of 5-year OS (*P* = 0.682) and DFS (*P* = 0.328). These results are comparable with those of previous studies. However, Sabbagh et al*.* reported that the 5-year OS (25% vs. 62%, *P* = 0.0003) and 5-year cancer-specific mortality (48% vs. 21%, *P* = 0.02) rates were significantly less favorable in the SEMS group, despite a greater number of resected lymph nodes (22 vs. 15, *P* = 0.002) compared with emergent surgery^41^. These differences could be explained by differences in the pathologic characteristics between the two groups. Tumor ulceration, peritumor ulceration, perineural invasion, and lymph node invasion were significantly more frequent in the stent group^42^.

There are several limitations to our study. First, as this study was performed retrospectively, selection bias may have occurred. Stent insertion may not be attempted in patients with complete obstruction because the procedure is very difficult. Moreover, during the preoperative evaluation period after stent insertion, the surgical plan may be altered to avoid elective surgery if the patient is at high risk for surgery. Additionally, ES could be performed in patients with unstable vital signs or conditions. Therefore, patients in the SEMS group might have had better perioperative outcomes than those in the emergent surgery group. Nevertheless, the patients in both groups had similar demographic characteristics and comorbidities. Moreover, although randomized clinical trials would be useful to establish guidelines for the treatment of colonic obstruction, several randomized controlled trials have been prematurely terminated due to unexpectedly high rates of stent-associated perforation (7–54%) and lower success rates (47–70%) without clinical benefits compared with emergent surgery^43–45^. Second, in this multicenter study, there were no specific guidelines for the treatment of colonic obstruction across the institutions. Instead, the treatment policy was based on the patient’s clinical status, as well as the physicians’ or surgeons’ preferences and experience across the six hospitals. However, despite the lack of treatment guidelines, our findings are consistent with the results of prior studies in terms of perioperative outcomes and oncologic outcomes. Third, despite being a multicenter study, stents were less frequently inserted in patients with right-sided cancers than left-sided cancers (14.8% vs. 85.2%). However, very few studies have compared stent insertion and emergent surgery in patients with right-sided obstructive CRC^16,35^. To show the results of stent insertion for both sides together, we tried to evaluate the safety and feasibility of SEMS in obstructive CRC regardless of the side of obstruction. Despite these limitations, the present study showed that SEMS has benefits for short-term perioperative outcomes, with similar long-term oncologic outcomes to those of ES, even when patients with right- and left-sided cancers were analyzed separately. Therefore, we believe that our results will provide valuable evidence for the management of obstructive CRC.

## Conclusion

The present study showed that SEMS insertion as a bridge to surgery was associated with faster recovery, a lower rate of stoma formation, and a higher rate of minimally invasive surgery, combined with similar oncologic outcomes to those of ES. Although certain technical difficulty and risk accompanied with stent insertion, SEMS insertion as a bridge to surgery could be considered as an alternative to ES for the management of patients with obstructive CRC.

## Data Availability

The datasets generated and/or analysed during the current study are available from the corresponding author on reasonable request.
